# Feasibility, acceptability and effect of the Mindful Practice curriculum in postgraduate training of general practitioners

**DOI:** 10.1186/s12909-021-02747-z

**Published:** 2021-06-07

**Authors:** Manuel Villarreal, Petra Hanson, Amy Clarke, Majid Khan, Jeremy Dale

**Affiliations:** 1 The Unit of Academic Primary Care, University of Warwick, Coventry, CV4 7AL UK; 2 Warwick Medical School, University of Warwick, Coventry, CV4 7AL UK; 3 School of Pharmacy, Centre for Behavioural Medicine, University College London, London, UK

## Abstract

**Background:**

Early career general practitioners are known to be at high risk of burnout. There is a need for widely applicable, cost-effective evidence-based interventions to develop trainees’ protective skills and strategies.

**Results:**

Of 120 eligible trainees, 23 (19.2%) expressed interest in participating, 17 subsequently started the course, and 15 completed at least 5 out of its 6 sessions. All psychological measures were stable for the six-week period prior to commencing the course. Following the course, there were statistically significant (*p* < 0.05) improvements in wellbeing, resilience, mindfulness, emotional exhaustion, disengagement, and stress scores. Participants described numerous benefits, and most stated that they would recommend it to colleagues.

**Conclusion:**

Including mindful practice within general practice vocational training is feasible, and in this study it benefited the psychological wellbeing of participants. Further research is needed to explore ways of increasing uptake and course completion, the sustainability of its effects, and the wider applicability of this approach.

## Introduction

General practitioners (GPs) at all stages of their career are at high risk of suffering from emotional stress, depression and burnout [1, 2], and this is contributing to increasing levels of sickness absence, and difficulties in retaining the GP workforce [3]. This is an international issue, with risk of burnout reflecting numerous factors including personal attributes, workload pressures, and difficult encounters with patients and colleagues [3, 4].

General Practice vocational training in the United Kingdom last 3 years and represents an opportunity for proactively equipping future GPs with effective coping skills [5]. This includes building resilience, the ability to bounce back or recover from stress [6]. However, despite recent interest in techniques to improve physicians’ resilience and wellbeing the evidence to support such interventions is sparse [7].

Mindfulness is the capacity for enhanced, non-judgmental and sustained moment-to-moment awareness of one’s own mental and emotional state and being, in the context of one’s immediate environment. There is evidence that mindfulness can improve doctors’ resilience, wellbeing, self-awareness and interpersonal skills [8–11], as well as patient-centred care [11–13]. Adapted mindfulness based programmes are becoming more popular in medical training, but requiring further assessment [14].

In a recent study with 47 GP trainees in the West Midlands, we demonstrated both a need and desire for greater wellbeing and resilience support as part of their vocational training [15]. On validated psychological scales, the trainees showed high prevalence for signs of burnout, including emotional disengagement (36; 80%) and exhaustion (35, 77%), with 29 (64%) scoring above the cut-off value for both. Over a third reported practising some form of mindfulness already, and most described interest in engaging in mindfulness practice. Specific work-related factors such as a lack of knowledge and training to deal with complex patients, inability to detach from work, and the need to keep up appearances, were described as factors that contributed to emotional exhaustion.

In the current study, we worked with the same cohort of trainees to explore the potential for incorporating mindfulness into their vocational training programme. We used the results of the previous study to adapt the Mindful Practice Curriculum (MPC) [16] for delivery as part of the vocational training programme. The MPC is training programme from the USA that has been specifically designed for physicians, and is based on Mindfulness-Based Stress Reduction (MBSR) [17]. It focuses on improving clinical resilience, quality of care and caring, and personal wellbeing; it appeared well-suited to addressing the challenges described by GP trainees.

The primary aim of the study was to assess the feasibility and acceptability of delivering the MPC training within the busy timetable for vocational training. Additionally, the impact of the MPC on participants’ psychological outcomes was explored, including well-being, disengagement, emotional exhaustion, resilience, stress management and mindfulness, together with participants’ views about the programme.

## Methods

### Recruitment of participants

All 120 Specialty Training (ST) trainee GPs from second year (ST2) and third year (ST3) in Coventry and Warwickshire were invited to participate in the programme. A presentation about the project was made at their weekly half-day release teaching, outlining the project’s aims and objectives, timescales, and details of the MPC. Following this, an email was sent to trainees, summarising the material that had been presented and providing a participant information sheet. It was explained that only those who could commit to attending at least five of the six sessions involved in the MPC were eligible to participate. Eligible participants completed informed consent.

### Intervention

The MPC aims to teach and reinforce patient-centered care through strengthening secular contemplative practices, narrative medicine, reflective questioning and appreciative inquiry. We adapted the programme to be delivered through weekly 1.5-h group sessions over a six-week period led by MK, a fully trained Mindful Practice tutor. Each session involved a didactic component in which information and research data relevant to the theme was presented, followed by a brief period of contemplative practice that included guided mindfulness practice and other exercises to practice at home and during clinical practice. Participants then engaged in a narrative exercise in which they were asked to recall a clinical experience related to the theme and were encouraged to write about their experience. These narrative exercises were used to share stories and practice using techniques of reflective questioning. The key themes covered in the course are described in Table 1. The sessions were delivered across lunchtime periods at a hospital teaching centre, which was felt to be the optimal time and location for enabling trainees’ participation.

**Table 1:** Course content

Themes	Methods
Professionalism	Reflective questioning. How do we learn? The difference between the ‘formal and informal’ curriculum. How do we deal with professional challenges? Some examples, and small group work
How Doctors think	Appreciative inquiry. Heuristics/ rational thinking. Factors that affect effective thinking. Biases: conscious and unconscious. An exploration of the factors that can influence a doctor’s decision-making ability
Witnessing suffering	What is suffering? An exploration of the causes/ consequences (both personal and professional), importantly in this session (although to an extent in every session), we look at the impact that suffering burnout and a lack of self-compassion has on the whole of our lives, not just the medical part)
Medical errors	Why do doctors make errors? What are typical responses to an error? What responses can be detrimental/ what other responses can be more skilful? An exploration of the impact (on the body/ mind and feelings) that errors has, as well as the subsequent impact on performance/ self-confidence. Alternative ways of handling/ responding to and dealing with medical errors are explored in smoke detail
Wellbeing and Burnout	How does burnout differ from depression? What are the causes and consequences? What are the thoughts / feelings and sensations associated with reduced/ reducing performance versus optimum performance? What are the workplace/ personal factors that contribute to this? What can we put in place to pre-empt and prevent?
Handling conflict compassionately	What makes a functional team? Personal attitudes/ beliefs/ attitudes of others? Are there signs that we are ‘heading towards’ conflict? What are these? How do they manifest in the body and the mind? How do we communicate this to others? How do we communicate with ourselves? To what extent a conflict with others a manifestation of conflict within us?

### Data collection

Data were collected via a range of validated instruments (see Table 2). Participants were invited via email to complete online questionnaires (using Qualtrics platform), including measures 6 weeks prior to the course, immediately prior to the course (to act as baseline measures), and up to 3 weeks after completion of the course. Participants were given a unique identifiable number, which allowed pairing of the responses immediately prior and post completion of the course; however, the unique identifier was not included with the measures completed 6 weeks prior to the course and so these scores could not be matched with the subsequent scores. Multiple reminders were sent to participants to encourage completion of the questionnaires.

**Table 2:** Validate psychological outcome measures that were used

Outcome measure	Assessed attribute	Scoring	Interpretation
The Oldenburg Burnout Inventory [18]	Burnout	Two components: emotional exhaustion and disengagement. Mean scores of each component are calculated (and reverse scoring applied when necessary).	Cut off scores of ≥2.25 for exhaustion and a score ≥ 2.10 for disengagement were used to predict problematic burnout [30], and burnout was indicated if both scores were above the given values.
Smith’s Brief Resilience Scale [6]	Resilience	Smith’s Brief Resilience scale consists of 6 items, 3 of which are reverse scored. The overall score is average of the six items.	1.0–2.99 indicate low resilience, 3.0–4.30 normal resilience and 4.31 to 5 high resilience
Cognitive and Affective Mindfulness Scale-Revised (CAMS-R) [19]	Mindfulness	10 items, with 6 being reversed scored. 4 response categories, from “Rarely/not at all” to “Sometimes” to “Often” to “Almost always”	The higher the score the higher mindful qualities.
Cohen’s Perceived Stress Scale [20]	Stress	10 items, with 5 responses from “Never” to “Almost never” to “Sometimes” to “Fairly often” to “Very often”, 4 reverse scoring	The higher the score, the more stress an individual is experiencing. Mean score for male is 12.1 and for female 13.7
Warwick-Edinburgh Mental Wellbeing Scale (WEMWS) [21]	Measure of mental well-being	14 items with 5 response categories, from “None of the time” to “All of the time”. Items are scored on a range from 1 to 5, providing a total score between 14 and 70.	WEMWBS score of less than 40 could indicate high risk of major depression and scores between 41 and 45 could be considered in high risk of psychological distress and increased risk of depression.

In addition, at the time of completion of the post-course questionnaire, participants answered questions related to acceptability and experience of the programme. This included barriers to attending the course, behavioural changes experienced, and overall views of the course.

In order to avoid potential concerns over confidentiality and to encourage openness, no personal data were collected.

### Data analysis

SPSS version 25 was used for data analysis. The analyses were planned on the assumption that data were normally distributed. Independent t-tests were used to analyse unmatched data (i.e. from data collected 6 weeks prior and immediately prior to the course delivery), and paired student t tests were used to analyse matched data (immediately prior and post course data). Intention to treat analysis was used for comparing results immediately prior and post course attendance. *P* < 0.05 was considered as a statistically significant different.

Qualitative data from the post-course questionnaire were analysed thematically.

### Ethical approval

Ethical approval was obtained from the University of Warwick’s Biomedical and Scientific Research Ethics Sub-Committee (REGO-2018-2292).

## Results

### Participants

**Fig. 1:**
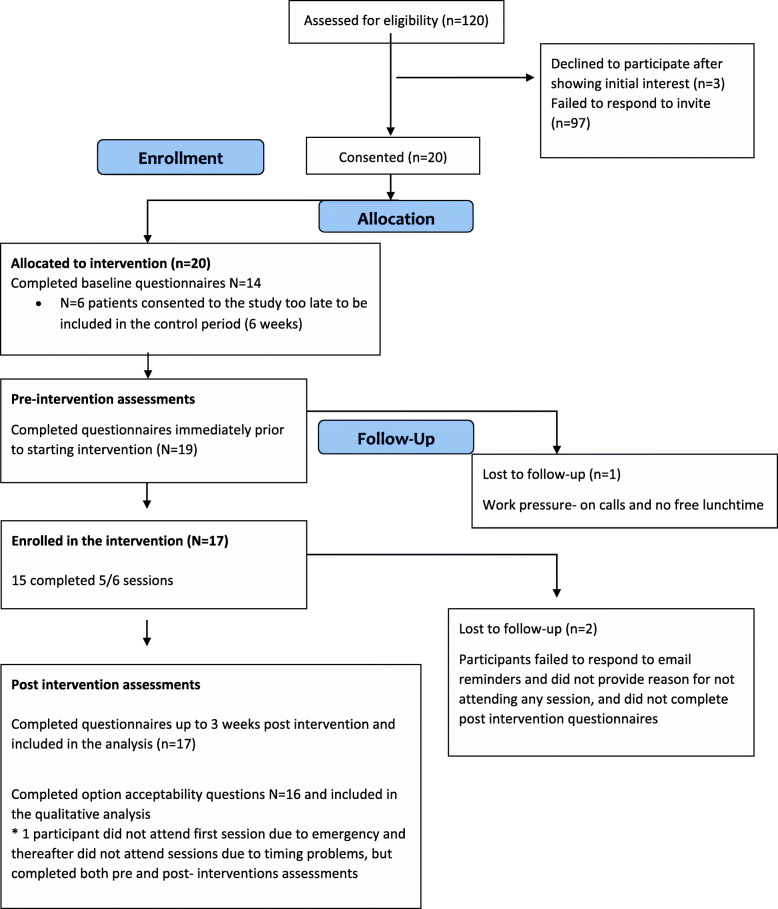
Consort diagram of participating flow

Twenty-three (19.2%) trainees stated that they could attend at least five of the six sessions and expressed interest in participating in the study, and 20 consented to take part. Of the latter, 17 subsequently started the course and 15 (88%) of them completed at least 5 out of its 6 sessions and were classed as “completers”. The other three consented individuals were unable to participate due to training schedule problems or changed personal circumstances. Consort diagram in Fig. 1 shows participant flow through the study.

Reasons for missing individual sessions mainly related to conflicting commitments and priorities within and outside work.

*“Though I used to start my surgery early to attend, seeing patient on home visit caused delay to come to course on time”. R9*

*“Difficulty with anything outside of the curriculum (especially as course running at same time as sitting exams) that it can feel like another extra thing”.R2*

*“It is hard to concentrate on the present when we have many worries.” R.5*

### Effect of MPC on psychological outcomes

Matched scores for pre- and post-course outcome measures were available for 17 participants. Fourteen had also completed outcome measures 6 weeks prior to the course.

Outcome scores obtained 6 weeks prior and immediately prior to the start of the course (Table 3) were stable, suggesting that levels of wellbeing, burnout, stress, and resilience, were consistent during this control period.

**Table 3:** Comparison of mean scores 6 week prior and immediately prior to intervention

	Mean Score 6 weeks prior to intervention (***N*** = 14)	Mean Score Immediately prior to intervention (***N*** = 19)	Mean difference	95% CI	***P*** value
**Wellbeing**	45.08	44.82	0.26	2.14 to −4.21	0.904
**Disengagement**	2.5	2.46	0.04	0.12 to −0.2	0.713
**Emotional exhaustion**	2.86	2.76	0.1	0.15 to −0.22	0.536
**Resilience**	3.1	3.02	0.08	−0.18 to 0.33	0.536
**Stress**	31.21	31.36	−0.15	2.78 to −3.08	0.918
**Mindfulness**	21.79	22.79	−1	−3.6 to 1.59	0.434

Following the course, there was significant improvement in all outcome measures (Table 4). For example, while 13 (76%) trainees scored above the threshold for significant disengagement (2.10) in the pre-course survey, this dropped to 5 (29%) participants post-course. There were 16 trainees (94%) who scored above the threshold (2.25) for emotional exhaustion pre-course, but only 9 (53%) post-course.

**Table 4:** Mean changes post course compared to immediately prior to course (*N* = 17)

	Mean Score immediately pre-intervention	Mean Score post-intervention	Difference post-pre	95%CI	***P*** value
**Wellbeing**	44.06	53.82	9.77	5.43 to 14.1	< 0.001
**Disengagement**	2.43	2.00	−0.43	−0.66 to − 0.2	0.001
**Emotional Exhaustion**	3.01	2.28	−0.73	−1 to − 0.45	< 0.001
**Resilience**	2.92	3.38	0.46	0.13 to 0.79	0.01
**Stress**	31.88	24.24	−7.65	−4.38 to-10.9	< 0.001
**Mindfulness**	22.65	28.65	6	2.28 to 9.72	0.004

### Participant experience

Most participants reported a positive outcome in their post-course feedback, mentioning improvement in coping with stress and feeling better able to manage emotional exhaustion.

*“I feel more able to recognize when I am tired and stressed”* R4

*“I feel I have tools to cope with stress and burnout better” R7*

The mindfulness skills gained through the course were generally felt to have been of value.

*“…being aware of your presence and surroundings, to observe rather than have a stress response”* R9

“…. *acknowledgment of feeling, body sensations and thoughts, including negative ones.” R13*

Only one participant did not feel the course had had any personal impact.

*“Unfortunately, not, there was no real new content for me and meditation was something I really don’t connect with” R14*

Most stated that they would recommend the course being made available to all doctors, with 15 out of 16 (93.8%) stating that they would like to see mindfulness training included as part of vocational training.

*“Absolutely, I think it could provide benefit to all trainees, a large part of our curriculum is on our fitness to practice with specific mention of our own health/ wellbeing and this course hugely supports this.”R4*

*“Absolutely. As a GP the public frustration about NHS seems to hit us hardest. It is a very demanding profession and without self-care it is extremely hard to be able to do this job until you retire.” R8*

However, there was recognition that the MPC course would not suit all trainees.

*“I don't think that all will get on board with this, therefore I don't think it would be worthwhile for all trainees. However, it has improved my resilience and I can see how it can reduce burnout etc*.”R18

## Discussion

### Summary

This study demonstrated the feasibility of delivering the six-week Mindful Practice Curriculum [16] as part of vocational training for general practice. The MPC was modified to fit within the constraints of general practice training in the UK and to introduce participants to a variety of topics that work synergistically to benefit wellbeing, resilience and burnout. Finding a convenient time and setting for the course delivery in the context of trainees’ busy schedules proved difficult. As participation in the programme was limited to trainees who could commit to attending at least five of the six sessions, those with conflicting clinical or training commitments, planned annual leave or other unavoidable personal issues were prevented from participating. However, almost all of those who did participate, completed it successfully and experienced positive personal gains. Compared to pre-course levels, there was statistically significant improvement in outcome measure scores in specific areas of resilience, mindfulness, stress and burnout. Almost all participants stated that they would like to see mindfulness training being incorporated into future vocational training and hence becoming accessible to all trainees.

### Strengths and limitations

The intervention drew on an evidence-based mindfulness training programme that has been specifically designed for doctors, and adapted it to increase its applicability and fit with the pressures of vocational training in the UK. Another strength was the use of validated psychological outcome measures. In the absence of a control group, the comparison of data at baseline and 6 weeks prior allowed us to demonstrate the persistent state of psychological health for the participants prior to participating in the course while the matching of pre- and post-course outcome measures enabled demonstration of the effect associated with course participation. Qualitative data collection provided greater depth to the findings, and understanding of how benefits were experienced; however, we lacked the resource to undertake a more in-depth qualitative exploration of participants’ experience, or of their intentions regarding future use of the skills that they have developed, limiting predictive sustainability of the programme.

The number of participants was sufficient to test the feasibility of intervention delivery as part of GP vocational training, but too small to draw definitive conclusions about its effectiveness. Despite the cohort of trainees who were eligible to participate in the course having high levels of stress and burnout and expressing a desire to engage in mindfulness training, as reported previously, relatively few volunteered to actually taking part. Finding a convenient and accessible time for delivering the course sessions was challenging, and individuals who were based in general practices that were distant from the hospital teaching centre found it more difficult to attend. Also, the need for participants to commit at the outset to attending at least 5 of the 6 sessions may have been off-putting for individuals who had annual leave or other conflicting commitments. For some individuals, the requirement to make an up-front commitment to attending the course may have been perceived as an addition stressor in the context of unavoidable everyday workload pressures. The novelty of this programme may have acted as an additional barrier for some individuals, and some may not have wanted to participate in research.

Given the small numbers involved and the lack of longer-term follow-up data, the effects observed on the measured psychological variables need to be interpreted with some caution. It was beyond the scope of the study to explore participants’ experience in-depth and how this affected outcomes; for example, participants may have varied in their interest in the weekly course topics (stress, burnout, recovery, concentration, well-being etc) and this may have affected the training effectiveness. In addition, longer term follow up is needed to demonstrate the sustainability of the skills gained beyond the period of the course itself.

### Comparison with existing literature

A recent systematic review summarised the evidence base for the positive impacts of Mindfulness-based interventions (MBIs) on doctors’ well-being and performance [22]. Several studies were reported as having methodological limitations due to self-selection of participants and lack of active control conditions. However, MBIs, similar to MPC, that included multiple essential mindfulness elements (development of greater attentional, emotional and behavioural self-regulation, as well as positive qualities such as compassion, wisdom, equanimity) in their content, or that employed a group-based training format, mostly showed positive effects [23–25].

While ours is the first study to focus on GP trainees using MPC and addressing specific needs and dynamics of physician’s activity, other MBIs have been shown to improve levels of mindfulness and reduce burnout in primary care professionals in Brazil [26], psychiatry trainees in Australia [27] and foundation trainee doctors and medical students in the UK [28, 29]. The latter study was an adaptation of the Mindfulness Based Cognitive Therapy (MBCT) programme for the workplace. A study from the USA with primary care staff (doctors, nurses, allied medical staff) indicated that the Mindfulness-based Wellness and Resilience (MBWR), another approach to mindfulness training, may be feasible and acceptable [30]. Recent study in France describes a protocol aiming to assess in a randomized control trial the long-term effectiveness and acceptability of a mindfulness-based intervention (MBI) compared with relaxation training (RT) [31].

### Implications for research and practice

There is an urgent need to promote wellbeing and resilience strategies within vocational training in order to prepare doctors for a career in general practice and lower the risk of emotional exhaustion and burnout in trainee and early career GPs [32]. This is important to retaining a healthy GP workforce, particularly given that relatively large numbers of trainees are currently intending to take career breaks, part-time work or leave the profession altogether [1, 2].

This study has demonstrated the feasibility within a real world context of delivering a six-week mindfulness course to trainee GPs. It found a high level of participant acceptability and identified associated benefits in participants’ mental wellbeing. While this needs to be interpreted in the context of this being a study conducted within one setting, with barriers such as competing vocational course priorities cited as a reason for a lack of engagement, the findings are sufficient to suggest that this offers a promising approach which may have widespread application. Further consideration is required of the resources that are needed to support the scaling up of this intervention, including protected time for undertaking wellbeing activities as part of vocational training, in order to support more widespread Integration into training programmes.

## Data Availability

Requests to access the data should be made to the corresponding author.
